# Nutritional Risk Assessment Scores Effectively Predict Mortality in Critically Ill Patients with Severe COVID-19

**DOI:** 10.3390/nu14102105

**Published:** 2022-05-18

**Authors:** Constantin Bodolea, Andrada Nemes, Lucretia Avram, Rares Craciun, Mihaela Coman, Mihaela Ene-Cocis, Cristina Ciobanu, Dana Crisan

**Affiliations:** 1 Intensive Care Unit, Clinical Municipal Hospital, 400139 Cluj-Napoca, Romania; cbodolea@gmail.com (C.B.); andrada.nemes@ymail.com (A.N.); mihaela_cocis@yahoo.com (M.E.-C.); eu_cristina_oana@yahoo.fr (C.C.); 2 Faculty of Medicine, “Iuliu Hațieganu” University of Medicine and Pharmacy, 400012 Cluj-Napoca, Romania; crisan.dc@gmail.com; 3 Department of Internal Medicine, Clinical Municipal Hospital, 400139 Cluj-Napoca, Romania; 4 Gastroenterology Clinic, “Prof. Dr. O. Fodor” Regional Institute of Gastroenterology and Hepatology, 400162 Cluj-Napoca, Romania; 5 Department of Medical Imaging, Clinical Municipal Hospital, 400139 Cluj-Napoca, Romania; mihaela_c87@yahoo.com

**Keywords:** malnutrition, nutritional risk, critical care, acute respiratory distress syndrome, COVID-19, mortality, intensive care

## Abstract

Background: Malnutrition predicts a worse outcome for critically ill patients. However, quick, easy-to-use nutritional risk assessment tools have not been adequately validated. Aims and Methods: The study aimed to evaluate the role of four biological nutritional risk assessment instruments (the Prognostic Nutritional Index—PNI, the Controlling Nutritional Status Score—CONUT, the Nutrition Risk in Critically Ill—NUTRIC, and the modified NUTRIC—mNUTRIC), along with CT-derived fat tissue and muscle mass measurements in predicting in-hospital mortality in a consecutive series of 90 patients hospitalized in the intensive care unit for COVID-19-associated ARDS. Results: In-hospital mortality was 46.7% (*n* = 42/90). Non-survivors had a significantly higher nutritional risk, as expressed by all four scores. All scores were independent predictors of mortality on the multivariate regression models. PNI had the best discriminative capabilities for mortality, with an area under the curve (AUC) of 0.77 for a cut-off value of 28.05. All scores had an AUC above 0.72. The volume of fat tissue and muscle mass were not associated with increased mortality risk. Conclusions: PNI, CONUT, NUTRIC, and mNUTRIC are valuable nutritional risk assessment tools that can accurately predict mortality in critically ill patients with COVID-19-associated ARDS.

## 1. Introduction

Since the human infection with the severe acute respiratory syndrome coronavirus-2 (SARS-CoV-2) was first documented in December 2019, the Coronavirus Disease 2019 (COVID-19) pandemic has brought the world to a halt, wreaking havoc on multiple fronts. According to the World Health Organization (https://covid19.who.int, accessed on 11 March 2022), as of March 2022, exactly two years after being classified as a pandemic, COVID-19 has claimed more than 6 million lives.

With particularities depending on the viral variant, COVID-19 was characterized by an extreme heterogeneity in potential disease forms. Therefore, the clinical phenotype ranged from asymptomatic and mildly symptomatic to very severe and critical, requiring intensive care unit (ICU) hospitalization, associated with high mortality [1]. From the early days of the pandemic, one primary research focus was identifying risk factors for poor outcomes. According to large-scale studies, the most common culprits were advanced age, cardiovascular disease, diabetes, underlying pulmonary disease, chronic kidney disease, and smoking [2,3]. Consequently, it appears to be a well-defined association between an overall frail state and COVID-19 related mortality.

One common finding in patients with a high comorbidity burden is a poor nutritional status. This might be a critical link in maintaining a disease-related vicious cycle, further perpetuating the consequences of chronic conditions and exacerbating the destabilizing effect of acute intercurrent disease, such as COVID-19 [4]. Pooled data from a meta-analysis including over 4000 patients hospitalized for COVID-19 has shown that up to 49% had evidence of malnutrition, assessed by various clinical tools, associated with significantly higher mortality [5]. Moreover, evidence suggests that prior history of malnutrition predisposes patients to severe COVID-19 in an age-dependent manner [6]. However, most of the pooled evidence is derived either from retrospective data based on medical coding or from studies with heterogeneous designs, mainly focusing on a single instrument analysis.

Nutritional risk is not only present in patients with predisposing risk factors, and, according to the European Society for Clinical Nutrition and Metabolism (ESPEN) guidelines on nutrition in the ICU, every critically ill patient staying in excess of 48 h in the ICU should be considered at risk for malnutrition [7]. There is a multitude of factors contributing to malnutrition in critically ill patients, ranging from an inflammation-induced hypercatabolic state to increased energy expenditure generated by ventilation, limited caloric intake, or muscle wasting due to physical immobilization [8,9]. Numerous tools can assess nutritional status, from anthropometrical measurements, to validated questionnaires, biological biomarkers, scores, and imaging-based methods [10]. However, in the context of patients already admitted to ICU, given the special conditions imposed by the pandemic, a comprehensive nutritional evaluation becomes even more difficult. While most ICU beds have integrated weight measurement tools, difficulties in interpreting body weight measurements due to potential fluid overload in various scenarios render actual weight prone to biases. Furthermore, other anthropometric measurements, impedance analysis, and dynamometer grip tests are difficult to perform in the setting of COVID-19. The logistic limitations typically encountered in the anthropometric evaluation of critically ill patients were thus further exacerbated [8].

Consequently, quick, easy-to-use, and readily available tools to assess nutritional status appear to be ever more crucial. Therefore, minimizing the additional steps required to evaluate risk might be relevant in day-to-day practice. Simple scores containing variables that are part of regular laboratory workups, and information derived from imaging regularly performed in all COVID-19 patients, such as thoracic CT scans, might significantly aid risk stratification for in-hospital adverse outcomes.

Among the widely validated easy-to-use instruments for nutritional assessment are the Prognostic Nutritional Index (PNI) [11], the Controlling Nutritional Status (CONUT) score [12], the Nutritional Risk in Critically Ill (NUTRIC) score [13], and the modified NUTRIC (mNUTRIC) score [14]. The variables required to compute the first two scores should be readily available in all hospital laboratories, as they only include serum albumin, total cholesterol, and lymphocyte count. NUTRIC requires interleukin-6 and the prior calculation of the APACHE II and the Sepsis-related Organ Failure Assessment (SOFA) scores, both frequently utilized in the ICU. To circumvent the need for interleukin-6, which might not be part of the biological armamentarium of each hospital, mNUTRIC excludes its value from the calculation formula, providing non-inferior risk assessment [14]. Indicators of malnutrition and sarcopenia derived from standard thoracic CT scans, such as fat tissue amount and disposition, muscle mass, or muscle density were also analyzed in the setting of COVID-19, with most protocols aiming to extrapolate data typically measured on abdominal imaging [15,16,17].

The current study aimed to evaluate the predictive capabilities of PNI, CONUT, NUTRIC, and mNUTRIC scores in predicting the need for mechanical ventilation and in-hospital mortality in patients with severe COVID-19 and acute respiratory distress syndrome (ARDS) admitted to the ICU.

As a secondary objective, the study assessed whether indicators of sarcopenia and poor nutritional status, as evidenced on standard thoracic CT scans, such as subcutaneous fat tissue, total intrathoracic fat tissue, and pectoral muscle mass and density, are associated with a worse prognosis.

## 2. Materials and Methods

### 2.1. Study Design and Participants

The current study’s design was retrospective, observational, longitudinal, and monocentric. A consecutive series of patients were enrolled from the ICU of a tertiary-care facility, serving as a high-volume, regional, COVID-19-dedicated center, between December 2020 and March 2021. On admission, the SARS-CoV-2 infection was documented by real-time reverse-transcriptase polymerase chain reaction (rRT-PCR). All of the included patients met the criteria for severe COVID-19 according to the thoracic CT scan. Patients with incomplete laboratory work-up or poor-quality thoracic CT-scan that prohibited an accurate assessment of the target variables were excluded. Patients admitted to the ICU for non-COVID-19-related causes, such as emergency surgical procedures, trauma, incidental findings of SARS-CoV-2 infection in patients with otherwise no COVID-19-related symptoms were excluded, as well as patients with no or mild lung involvement.

### 2.2. Baseline Evaluation

Patient demographics and complete history were recorded on admission. The Charlson comorbidity index was used to facilitate a standardized approach to underlying disease burden, and it was calculated on admission, as initially described by Charlson M et al. in 1987 [18]. The complete laboratory workup was recorded within the first 24 h of hospital admission, including the following variables: complete blood count, inflammation markers (C-reactive protein—CRP, procalcitonin), coagulation (prothrombin time—PT, international normalized ratio—INR, activated partial thromboplastin time—aPTT, fibrinogen, D-dimers), metabolic profile (fasting blood glucose, serum triglyceride levels, total cholesterol, HDL, LDL cholesterol, total protein levels, and serum albumin), kidney function (urea and serum creatinine), electrolytes (sodium and potassium), liver function tests (aspartate aminotransferase—AST, alanyl aminotransferase—ALT, total and direct bilirubin, GGT—gamma-glutamyl transferase, and alkaline phosphatase—ALP), and N-terminal pro B-type natriuretic peptide (NT-proBNP) as a biomarker for heart failure. Interleukin-6 (IL-6) was recorded within 24 h of ICU admission. The PaO_2_/FiO_2_ ratio was registered at the time of ICU admission.

### 2.3. Scores and Indexes

The APACHE II and Sepsis-related Organ Failure Assessment (SOFA) scores were calculated on ICU admission, according to their original description [19,20]. The NUTRIC and modified NUTRIC (mNUTRIC) scores were calculated within the first 24 h of ICU admission. PNI and CONUT were calculated on hospital admission. The calculation formulas, as well as their original description are depicted in Table 1.

### 2.4. Imaging

A standard native thoracic CT scan was performed within the first 24 h of hospitalization using two multidetector CT scanners: 20-slice Somatom Definition AS and 32-slice Somatom go.Up (Siemens Healthineers, Erlanger, Germany). COVID-19 disease severity was assessed using the total severity score (TSS), as described by Li K et al. [21]. The score quantitatively assesses the acute inflammatory lesions in each pulmonary lobe, attributing to each lobe a score ranging from 0—none to 4—exceeding 75% of lobar parenchyma, leading to a maximum total score of 20. According to the original description, a minimum score of 8 defines severe disease. Some of the patients had multiple re-evaluations based on the disease course, and the maximum TSS was recorded. A single radiologist has reinterpreted all thoracic CT scans to avoid inter-observer variability for the study.

**Figure 1:**
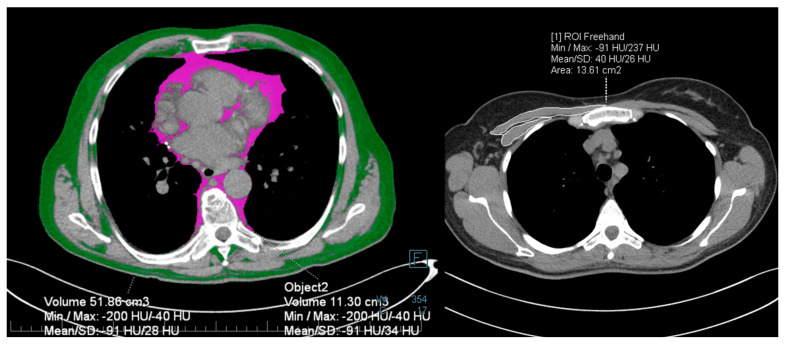
Thoracic fat and pectoralis muscle measurement. Intrathoracic fat (magenta) and thoracic subcutaneous fat (green) outlined at the level between the seventh and eighth thoracic vertebrae (**left**). Pectoralis muscle area outlined in white (**right**) at transverse section situated cranially from the aortic arch.

The index thoracic CT scan was used to measure the volume of subcutaneous fat, intrathoracic fat, total fat, pectoral muscle area, and pectoral muscle density. A single radiologist performed the measurements, using the syngo.via VB30A imaging software (Siemens Healthineers, Erlanger, Germany). A single 3-mm slice situated between the seventh and eighth thoracic vertebrae was used to quantify total thoracic and intrathoracic adipose tissue, adapting from a previously described method [22]. A region of interest of the thoracic circumference was contoured manually, and a density range from −200 to −40 HU (Hounsfield Unit) was applied, obtaining the total thoracic fat volume expressed in mm. Intrathoracic fat volume was outlined similarly on the same slice (Figure 1). Extrathoracic adipose tissue volume was obtained by subtracting total thoracic fat and intrathoracic fat. Sarcopenia was evaluated using the pectoralis muscle cross-sectional area (PMA) and mean muscle density (HU). An axial slice located cranially to the aortic arch was selected, applying the density range of −50 to 142 HU. The pectoralis major and minor borders on the right side were manually outlined (Figure 1). To account for the physiological fat-tissue distribution disparity between genders, a separate subgroup analysis was performed for fat-tissue and muscle assessment.

### 2.5. Statistical Analysis

A certified biomedical statistician designed and performed the complete analysis using the SPSS 28.0 software (SPSS Inc., Chicago, IL, USA). Continuous variables were described and analyzed according to the normality of their distribution. The Shapiro–Wilk test was used to assess the normality of the distribution. Normally distributed variables were expressed as mean ± standard deviation (SD) and compared using the Student’s *t*-test. Skewed variables were expressed as median and 95% confidence interval (CI) and compared using the Mann–Whitney U test. Categorical variables were analyzed using the chi-square test. The statistical significance threshold was 0.05. The Cox proportional hazards model was used to analyze the association between overall in-hospital survival or event-free survival (mechanical ventilation) and the variables of interest. The multivariate analysis was designed to avoid model overfitting and multicollinearity. Consequently, variables with similar computational formulas associated with an outcome on univariate Cox regression were not included in the same multivariate analysis. Therefore, multiple multivariate scenarios were designed. The discriminative performance of target variables was evaluated using the area under the receiver operating characteristic (AUROC) analysis.

### 2.6. Ethical Considerations

The Cluj-Napoca Clinical Municipal Hospital’s Ethics Committee has approved the current research protocol. The study protocol complied with the ethical guidelines of the modified 1975 Declaration of Helsinki. Informed consent was obtained from all subjects involved in the study. Patient-related data were managed according to the European Union General Data Protection Regulation (GDPR).

## 3. Results

Ninety consecutive patients with severe COVID-19 and ARDS met the inclusion criteria of our protocol. The median age was 67 (range 36–89), and the percentage of male patients was 58.9% (*n* = 53). The rate of mechanical ventilation was 49.4% (*n* = 44) and in-hospital mortality was 46.7% (*n* = 42). The entirety of the relevant baseline characteristics in the entire group as well dichotomized based on survival status, is depicted in Table 2.

There were no significant discrepancies between groups regarding the extent of COVID-19-related lung involvement. Patients who survived had a significantly lower median age, had a shorter ICU stay, had better ventilation parameters, and had a lower APACHE II score, suggestive of a better overall shape at the time of ICU admission. A significantly higher proportion of patients requiring mechanical ventilation was among the non-survivors, as 88.1% (*n* = 37/42) were eventually intubated, compared to 16.6% for survivors. However, 23 of the 42 patients (54.7%) were intubated in the 24 h before their death for various reasons, as is frequently the case for critically ill patients with multi-systemic organ failure, significantly skewing the proportion of patients requiring mechanical ventilation towards the deceased group.

Non-survivors were at a significantly higher nutritional risk on all four nutritional assessment scores included in the study, namely PNI, CONUT, NUTRIC, and mNUTRIC. There were no differences regarding the amount of fat tissue measured on the thoracic CT scan, nor regarding PMA or PM density. A subgroup analysis was performed to account for the potential gender-related discrepancies determined by muscle and fat tissue disposition. There were no significant differences between groups regarding imaging-based variables, and none were associated with either an increased risk for mechanical ventilation or in-hospital mortality.

The predictive capabilities for the risk of mechanical ventilation and in-hospital mortality of the variables with significant median differences between survivors and the deceased were analyzed using a univariate Cox proportional hazards model and are depicted in Table 3.

None of the biological variables (albumin, NT-proBNP, or interleukin-6) were associated with an increased risk of mechanical ventilation when analyzed independently. Among the nutritional assessment tools, only NUTRIC and mNUTRIC were significantly associated with an increased risk of mechanical ventilation, while PNI and CONUT, which include metabolic variables (albumin and cholesterol), were not.

Given that the variables significantly associated with outcomes had a substantial overlap regarding the computing parameters, we could not perform an adequate multivariate analysis with all significant predictors on univariate analysis due to extreme model overfitting. For example, APACHE II and interleukin-6 are included in the NUTRIC and mNUTRIC scores; multiple scores include age; and albumin is a crucial determinant in some nutritional risk assessment scores. Therefore, we designed various multivariate analysis scenarios for the risk of in-hospital death to avoid collinearity, depicted in Table 4. The first two scenarios revealed that PNI and CONUT, which do not take patient history (Charlson Index) and current state of homeostasis (APACHE II) into account, can independently predict survival. The following two scenarios evaluated the independent predictive capabilities of NUTRIC and mNUTRIC, respectively, when compared to the Charlson Comorbidity Index. Both scores have retained their statistical significance in these scenarios.

**Figure 2:**
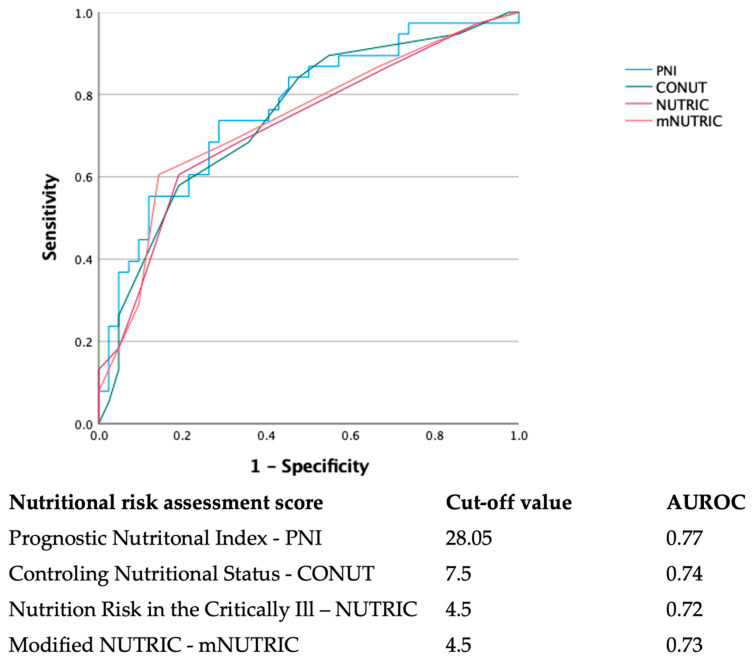
The AUROC curves of the nutritional risk assessment scores for predicting in-hospital mortality.

We performed an AUROC analysis to evaluate the discriminative capabilities of PNI, CONUT, NUTRIC, and mNUTRIC for in-hospital mortality. While all four scores had shown good discriminating capabilities, PNI had the largest AUROC, at 0.77, with the best cut-off value of 28. NUTRIC and mNUTRIC had similar discriminatory prowess, with a slightly larger AUROC for mNUTRIC, potentially negating the added value of interleukin-6 in the computation formula of NUTRIC. The AUCs for each score and their respective metrics are depicted in Figure 2. Of note, the AUCs for APACHE II and the Charlson Comorbidity index in our dataset were 0.69 (for an optimal cut-off of 15.5) and 0.74 (cut-off of 3.5), respectively.

Combining the scores that independently predicted mortality on multivariate analysis did not lead to statistically significant improvements in discrimination. Therefore, combining APACHE II and PNI has improved AUC by 0.035 (*p* = 0.305), while combining APACHE II and CONUT has improved AUC by 0.048 (*p* = 0.179).

## 4. Discussion

In response to its primary objective, our study has shown that the non-invasive nutritional assessment of patients with severe COVID-19 admitted in the ICU, using the PNI, CONUT, NUTRIC, and mNUTRIC, effectively predicts in-hospital mortality. Thus, patients with a higher nutritional risk expressed by the above scores had a significantly worse outcome. Moreover, these scores had at least similar prognostic capabilities to the more established predictive systems frequently used in intensive care, such as the APACHE II and SOFA scores. Regarding the secondary objectives of our research, data extracted from a native thoracic CT scan did not provide relevant prognostic information, as differences in thoracic fat tissue amount, disposition, muscle density, or PMA were not associated with a worse outcome.

Our results align with most of the previously published articles in the field regarding the role of biological indexes in predicting the outcomes of critically ill patients. However, data regarding their role in the setting of patients with ARDS or COVID-19 is relatively scarce. Furthermore, there is little evidence regarding any comparisons between biomarkers for nutritional risk and other imaging-derived instruments.

A Japanese study group initially developed the PNI to predict the surgical risk associated with malnutrition in gastric cancer patients [11]. Since its initial validation, its predictive capabilities have been documented in various clinical scenarios, including oncology [23], cardiology [24], and critical care [25]. In the setting of COVID-19, previously published articles have reported that a lower PNI score was associated with a more severe disease form [26,27] and higher COVID-19-related mortality [28,29,30]. The two previously reported cut-off values for predicting mortality were slightly discrepant, as one study suggested a cut-off below 33.4 [28], and the other 42 [30]. Our study’s cut-off value with the best discriminative capabilities (AUROC of 0.77) was 28, significantly lower than the other values. The discrepancies in cut-off values might result from each study’s relatively small sample sizes and the different study populations (East-Asian, West-Asian, and Central European, respectively).

The CONUT score was initially developed in 2005 as an easy-to-use substitute for a Full Nutritional Assessment [12]. CONUT categorizes patients into four states depending on the score (range 0–12), from normal to severe nutritional imbalance. Like PNI, CONUT was validated in a wide range of clinical scenarios and was associated with many ICU-related outcomes, from incidence of delirium [31] to mortality in various conditions such as trauma, heart failure, or irrespective of ICU-admittance cause [32,33,34]. While to this point there have been no published articles on the relevance of nutritional evaluation using CONUT in COVID-19 patients hospitalized solely in the ICU, the available data suggests that patients with a higher CONUT score were prone to more severe outcomes, having a more extended hospital stay, required mechanical ventilation more frequently, and had higher in-hospital mortality [35,36,37]. In a study including patients across the entire COVID-19 disease severity spectrum, a CONUT score above 5.5 predicted mortality with an AUROC of 0.83, suggesting that even a mild-to-moderate nutritional imbalance might predispose patients to a worse disease trajectory [36]. Our cut-off of 7.5 was significantly higher, placing most of the patients exceeding this cut-off value in the severe malnutrition group. However, in a study including 1627 COVID-19 patients irrespective of disease severity, only 4.9% were classified as severely malnourished based on the CONUT score [35], compared to 25.5% among the patients with severe COVID-19 in our cohort. This might be explained by the fact that patients with a higher degree of nutritional imbalance are more prone to being admitted to the ICU, thus potentially skewing the cut-off towards higher values.

Similar to PNI and CONUT, the reports on NUTRIC and mNUTRIC in COVID-19 have been scarce. If the PNI and CONUT were initially designed for oncological patients and the general population, respectively, the NUTRIC score was specifically designed for the nutritional evaluation of patients expected to stay more than 24 h in the ICU [13]. In response to the relatively low availability of IL-6 in day-to-day practice, Rahman A et al. have proposed an abbreviated version of the score, mNUTRIC, which does not require IL-6 and has been extensively validated since the first report in 2016 [14]. Regarding the NUTRIC and mNUTRIC in severe COVID-19 requiring ICU admission, all reports were concordant, suggesting that higher scores accurately predict higher mortality [38,39,40,41]. Furthermore, there is evidence that mNUTRIC effectively predicts mortality in other clinical entities associated with acute respiratory failure, such as community-acquired pneumonia [42]. Our findings follow the same trendline, as deceased patients had significantly higher NUTRIC and mNUTRIC scores. Furthermore, according to our dataset, there was no substantial gain in the predictive prowess due to the use of IL-6, as mNUTRIC even had slightly better discrimination metrics, with an AUROC of 0.73 versus 0.72 for NUTRIC, using the same cut-off value of 4.5.

While some studies have been published since the pandemic’s outburst on the role of individual nutritional risk assessment tools, most have focused on a single non-invasive score or index. Thus, one of the strengths of our design is the multitude of scores analyzed, supporting the need for nutritional evaluation regardless of the instrument of choice, as PNI, CONUT, NUTRIC, and mNUTRIC were similarly effective predictors. The main advantage of using these tools is that they do not require anamnestic or hetero-anamnestic data, which are especially prone to bias in an acute critical condition. In contrast to other well-established criteria for malnutrition, such as GLIM, neither data regarding prior non-volitional weight loss or dietary intake nor cumbersome anthropometric measurements are needed [43]. Furthermore, it is worth discussing the diminished value of PNI and CONUT for predicting the risk for mechanical ventilation, compared to the risk of in-hospital stays. One potential explanation might be that death in patients with a high-nutritional risk might not be closely tied to the extent of respiratory failure, but rather due to a higher risk for any of the other various complications that can occur in critically ill patients. In contrast, NUTRIC and mNUTRIC were effective in predicting the need for mechanical ventilation, which might be explained by incorporating the APACHE II and SOFA scores in their computational formula.

Moreover, our findings suggest that indicators for malnutrition might be more subtle in critical patients, as biological variables appear to predict nutritional status better than gross anthropometric variables and quantitative imaging-based methods. In the setting of COVID-19, it became evident that fat tissue assessment and the analysis of muscle mass, typically analyzed in the lumbar region on abdominal CT scans, must be validated with surrogates on thoracic CT acquisitions, since this quickly became the gold standard to assess COVID-19 disease severity. One report from a Dutch study group has indicated that skeletal muscle mass determined on a thoracic CT scan appeared to correlate well to measurements on the lumbar region on scans belonging to the same patient [15]. However, the role of various fat-tissue and muscle mass measurements on thoracic CT scans in predicting a worse outcome has yet to be clearly defined. An article published by Schiaffino S et al. has indicated that a low T5 paravertebral muscle area was associated with a significantly higher risk of ICU admission or death (OR 2.3, 95% CI 1.3–3.7) on a cohort exceeding 500 patients [16]. However, these findings have not been replicated by a Mexican study group in a similar-sized cohort, which found no evidence of an increased risk for ICU admission, mechanical ventilation, or in-hospital mortality for patients with low T12 skeletal mass index [17]. Another small-scale study including 67 patients with severe COVID-19 reported that a low thoracic muscle cross-sectional area, PMA, and high intra-thoracic fat tissue were associated with a higher mortality [44]. We found no association between PMA and worse outcomes. Nevertheless, contrary to some aforementioned studies [16,17], our protocol included only patients with severe COVID-19 and ARDS, thus selecting patients who were already prone to a worse outcome than the general population. Thus, our design did not account for the risk of ICU admission and an implicit higher risk of mortality in a general COVID-19 population, potentially negating a predictive effect. Our data did not reveal any gender discrepancies regarding fat tissue disposition, muscle mass, or muscle density.

The main limitation of our design was its observational nature, as assessing the role of nutritional intervention to improve outcome would have significantly increased the value of our findings. Moreover, none of the variables included in our study have been internally validated against other extensive nutritional assessment tools. However, according to the latest ESPEN guidelines on clinical nutrition in the ICU, published in 2019, there is no consensus on the superiority of any given instrument, as a 100% consensus recommendation states that “A general clinical assessment should be performed to assess malnutrition in the ICU, until a specific tool has been validated” [7].

The latter statement further emphasizes the dire need for evidence regarding nutritional risk evaluation in all clinical scenarios involving critically ill patients, as nutritional risk appears to be a significant outcome predictor in the ICU. Moreover, given the particularities of patient care in the ICU, which sometimes might include insufficient data on patient history or prior states, difficulties in evaluating muscle strength, or the lack of prioritization of anthropometric measurements, which are time-consuming, the focus should be on quick easy-to-use tools that require as little additional steps as possible.

## 5. Conclusions

Non-invasive biological scores for nutritional risk assessment tools such as PNI, CONUT, NUTRIC, and mNUTRIC are effective independent predictors for the risk of in-hospital mortality in patients with severe COVID-19 and ARDS hospitalized in the ICU. They appear to have a higher prognostic value compared to measurements derived from a standard thoracic CT scan.

## Figures and Tables

**Table 1:** Scores and indexes utilized in the study.

Score	Abbreviation	Variables/Calculation Formula	Original Reference
Prognostic Nutritional Index	PNI	(10 × serum albumin (g/dL)) + (0.005 × lymphocytes/μL)	Onodera T, et al. [11]
Controlling Nutritional Status	CONUT	Point scoring based on serum albumin (g/dL), lymphocyte count/mL, and total cholesterol (mg/dL), ranging from 0–1 (normal), 2–4 (mild), 5–8 (moderate), and 9–12 (severe)	Ignacio de Ulíbarri J, et al. [12]
Nutritional Risk in Critically Ill	NUTRIC	Age, APACHE II, SOFA scores, number of comorbidities, days in hospital to ICU admission, and Interleukin-6	Heyland D, et al. [13]
Modified Nutritional Risk in Critically Ill	mNUTRIC	Age, APACHE II, SOFA scores, number of comorbidities, and days in hospital to ICU admission	Rahman A, et al. [14]

**Table 2:** Baseline characteristics of the study population and comparison between groups based on survivor status.

Variable	Entire Group (*n* = 90)	Survivors (*n* = 48)	Deceased (*n* = 42)	*p*-Value
General data				
Age (years)	67 (63.2–67.9)	62.5 (57.9–64.1)	72.5 (67.7–73.7)	<0.001
Gender, male (*n*, %)	53 (58.9%)	26 (54.1%)	27 (64.2%)	0.334
Charlson Comorbidity Index	4 (4–5.4)	3 (2.8–4.6)	5 (5–6.7)	<0.002
Total hospital stay (days)	24 (23.8–31.2)	29.5 (24.4–35.3)	21.5 (19.8–29.8)	0.139
Length of ICU stay (days)	11.1 (11–17.1)	8 (7.8–16.2)	14 (12–20.9)	<0.001
Mechanical ventilation (*n*, %)	45 (50%)	8 (16.6%)	37 (88.0%)	<0.001
PaO_2_/FiO_2_ at ICU admission	116 (112.1–142)	133.5 (128–165.7)	87.5 (82.2–126.1)	0.022
SOFA score at ICU admission	5 (4.8–6.1)	4 (3.9–5.3)	5.5 (5.5–7.1)	0.079
APACHE II score at ICU admission	15 (14.1–17.3)	14 (11.2–15.3)	17.1 (16.1–21.1)	0.028
Laboratory work-up				
Hemoglobin (g/dL)	13.8 (13–13.9)	14 (13.3–14.3)	13.6 (12.4–13.7)	0.294
White blood cell count (×10^9^/L)	7.1 (6.8–9.6)	7.6 (7.6–10.4)	6.8 (6.7–9.8)	0.520
Neutrophil count (×10^9^/L)	5.8 (5.5–8.2)	6.2 (6.0–8.8)	5.4 (5.3–8.3)	0.131
Lymphocyte count (×10^9^/L)	0.8 (0.8–1.1)	0.86 (0.82–1.22)	0.79 (0.71–1.15)	0.837
Platelet count (×10^9^/L)	193 (192–238.2)	193 (188.2–249.5)	193.5 (176.8–246.8)	0.982
C-reactive protein (mg/dL)	14 (12–16.9)	15 (119.2–190.1)	9.6 (9.1–15.6)	0.599
Procalcitonin (ng/dL)	0.1 (0.0–3.3)	0.1 (0.0–3.6)	0.1 (0.0–3.1)	0.741
Interleukin-6 (pg/mL)	23.1 (20–205.2)	13 (11.1–104.2)	33.8 (11.2–289.2)	0.040
Creatinine (mg/dL)	1.1 (1–1.9)	1.0 (0.9–1.7)	1.2 (1.1–2.6)	0.063
NT-proBNP (pg/mL)	506 (302.2–4 560.1)	361.8 (20.6–3062.1)	830 (760.2–7458.5)	0.033
Albumin (g/dL)	2.9 (2.8–3)	3.1 (3–3.2)	2.8 (2.6–2.9)	<0.001
Total protein (g/dL)	5.4 (5.2–5.8)	5.4 (5.4–5.8)	5.3 (4.9–6.1)	0.891
Cholesterol (mg/dL)	138 (132.6–152)	145.5 (135.1–159.7)	134 (120.8–152.2)	0.551
Triglycerides (mg/dL)	158 (152–207.2)	164 (148.7–212.4)	151.5 (145.5–224.2)	0.526
Imaging				
TSS at admission	14 (12–14.1)	13 (11.4–14.1)	14 (11.7–14.7)	0.899
Peak TSS during hospital stay	17 (14.7–17.3)	15 (13.4–16)	18 (15.4–17.6)	0.062
Subcutaneous fat (cm^3^)	77.9 (70.9–94.5)	88.2 (80.1–105.5)	67.2 (66.6–89.6)	0.062
Intrathoracic fat (cm^3^)	9.8 (9.5–11.6)	10.6 (9.4–12.5)	9 (8.8–11.5)	0.131
Total fat (cm^3^)	84.5 (88–105.5)	96.7 (91.9–117.1)	76.8 (76.1–99.1)	0.131
Pectoralis muscle area (cm^2^)	18.9 (18.1–20.9)	19.1 (17.5–21.4)	18.7 (17.6–21.6)	0.835
Pectoralis muscle density (HU)	18.5 (16.1–21.2)	18.5 (15.7–22.8)	18.5 (14–21.3)	0.834
Nutritional risk assessment scores				
PNI	30 (28.5–30.5)	31.5 (30.2–32.6)	28 (26–28.9)	<0.001
CONUT	7 (6.1–7.2)	5 (4.9–6.3)	8 (7–8.6)	0.010
NUTRIC	3.5 (3.5–4.3)	3 (2.7–3.7)	5 (4.1–5.4)	<0.001
mNUTRIC	3 (2.9–4.2)	3 (2.7–3.6)	5 (4–5.2)	<0.001

All variables had a non-normal distribution and were expressed as median and 95% confidence interval. ICU—intensive care unit; NT-proBNP—N-terminal pro B-type natriuretic peptide; TSS—COVID-19 total severity score; HU—Hounsfield units; PNI—prognostic nutritional index; CONUT—controlling nutritional status score; NUTRIC—nutrition risk in critically ill score; and mNUTRIC—modified nutrition risk in critically ill.

**Table 3:** Univariate Cox proportional hazards analysis for the risk of mechanical ventilation and in-hospital mortality.

	Mechanical Ventilation			In-Hospital Mortality		
Variables	Hazard Ratio	95% Confidence Interval	*p*-Value	Hazard Ratio	95% Confidence Interval	*p*-Value
Age (years)	1.03	1.00–1.06	0.048	1.05	1.02–1.09	<0.001
Charlson Comorbidity Index	1.03	0.95–1.12	0.362	1.09	1.01–1.18	0.033
PaO_2_/FiO_2_ at ICU admission	0.99	0.98–1.00	0.271	0.99	0.99–1.00	0.621
APACHE II score	1.08	1.03–1.12	0.004	1.07	1.02–1.11	0.003
Interleukin-6 (pg/mL)	1.000	0.992–1.000	0.918	1.000	0.991–1.000	0.911
NT-proBNP (pg/mL)	1.000	1.000–1.001	0.943	1.000	1.000–1.001	0.322
Albumin (g/dL)	0.96	0.90–1.02	0.332	0.91	0.85–0.98	0.012
Subcutaneous fat (cm^3^)	0.99	0.99–1.00	0.531	0.99	0.98–1.00	0.094
PNI	0.96	0.90–1.03	0.333	0.91	0.85–0.98	0.011
CONUT	1.05	0.94–1.18	0.303	1.15	1.02–1.29	0.014
NUTRIC	1.27	1.07–1.51	<0.001	1.31	1.10–1.56	<0.001
mNUTRIC	1.30	1.10–1.54	<0.001	1.37	1.15–1.62	<0.001

ICU—intensive care unit; NT-proBNP—N-terminal pro B-type natriuretic peptide; PNI—prognostic nutritional index; CONUT—controlling nutritional status score; NUTRIC—nutrition risk in critically ill score; and mNUTRIC—modified nutrition risk in critically ill score.

**Table 4:** Multivariate Cox proportional hazards analysis for the risk of in-hospital mortality.

Variables	Hazard Ratio	95% Confidence Interval	*p*-Value
Scenario 1			
Charlson Comorbidity Index	1.04	0.95–1.15	0.310
APACHE II	1.06	1.02–1.11	<0.001
PNI	0.93	0.87–0.98	0.041
Scenario 2			
Charlson Comorbidity Index	1.02	0.93–1.13	0.571
APACHE II	1.07	1.02–1.12	<0.001
CONUT	1.14	1.03–1.30	0.050
Scenario 3			
Charlson Comorbidity Index	1.05	0.96–1.16	0.221
NUTRIC	1.28	1.07–1.54	<0.001
Scenario 4			
Charlson Comorbidity Index	1.05	0.96–1.16	0.252
mNUTRIC	1.33	1.12–1.59	<0.001

PNI—prognostic nutritional index; CONUT—controlling nutritional status score; NUTRIC—nutrition risk in critically ill score; and mNUTRIC—modified nutrition risk in critically ill.

## Data Availability

Not applicable.
